# Effect of N Fertilization Pattern on Rice Yield, N Use Efficiency and Fertilizer–N Fate in the Yangtze River Basin, China

**DOI:** 10.1371/journal.pone.0166002

**Published:** 2016-11-18

**Authors:** Xiaowei Liu, Huoyan Wang, Jianmin Zhou, Fengqin Hu, Dejin Zhu, Zhaoming Chen, Yongzhe Liu

**Affiliations:** 1 State Key Laboratory of Soil and Sustainable Agriculture, Institute of Soil Science, Chinese Academy of Sciences, Nanjing, P.R. China; 2 University of Chinese Academy of Sciences, Beijing, P.R. China; 3 Agriculture Committee of Jiangyan City, Taizhou, 225599, P.R. China; 4 Ministry of Agriculture, Nanjing Agricultural University, Nanjing, P.R. China; University of Delhi, INDIA

## Abstract

High N loss and low N use efficiency (NUE), caused by high N fertilizer inputs and inappropriate fertilization patterns, have become important issues in the rice (*Oryza sativa* L.) growing regions of southern China. Changing current farmer fertilizer practice (FFP, 225 kg ha^–1^ N as three applications, 40% as basal fertilizer, 30% as tillering fertilizer and 30% as jointing fertilizer) to one—time root—zone fertilization (RZF, 225 kg ha^–1^ N applied once into 10 cm deep holes positioned 5 cm from the rice root as basal fertilizer) will address this problem. A two—year field experiment covering two rice growing regions was conducted to investigate the effect of urea one—time RZF on rice growth, nutrient uptake, and NUE. The highest NH_4_^+^–N content for RZF at fertilizer point at 30 d and 60 d after fertilization were 861.8 and 369.9 mg kg^–1^ higher than FFP, respectively. Rice yield and total N accumulation of RZF increased by 4.3–44.9% and 12.7–111.2% compared to FFP, respectively. RZF reduced fertilizer—N loss by 56.3–81.9% compared to FFP. The NUEs following RZF (mean of 65.8% for the difference method and 43.7% for the labelled method) were significantly higher than FFP (mean of 35.7% for the difference method and 14.4% for the labelled method). In conclusion, RZF maintained substantial levels of fertilizer—N in the root—zone, which led to enhanced rice biomass and N uptake during the early growth stages, increased fertilizer—N residual levels and reduced fertilizer—N loss at harvest. RZF produced a higher yield increment and showed an increased capacity to resist environmental threats than FFP in sandy soils. Therefore, adopting suitable fertilizer patterns plays a key role in enhancing agricultural benefits.

## Introduction

Rice is an important staple food crop for more than 3 billion people in the world and for about 60% of the Chinese population [1]. Increase in rice production is needed if rising population demand is to be met. However, cultivable natural land resources are limited. Therefore, much of this increase must come from improved yield per hectare. Nitrogen (N) is one of the main factors affecting rice yield and has been excessively applied in China [2]. The Chinese national average N application rate for rice increased from 145 kg N ha^–1^ in 1997 to 300 kg N ha^–1^ in 2006, which is significantly higher than the global average. It even reached 360 kg N ha^–1^ in the Taihu lake region [3]. Three splits of N fertilizer (FFP, one basal fertilizer and two top—dressings) is the conventional fertilization method for rice, and the basal fertilizer application is usually applied by broadcasting. Higher farm subsidies and lower N fertilizer prices have further increased N inputs [4]. Inappropriate fertilization patterns and excessive use of N fertilizer have resulted in considerable N losses through ammonia (NH_3_) volatilization and leaching [5, 6]. This has meant that NUE has been as low as ~35% (15–20% lower than other major rice growing countries) [6].

Some effective measures have been recommended for lowering N rate and enhancing NUE, such as applying N at a later growth stage [5], adjusting the N rate based on chlorophyll readings [7], applying controlled release N fertilizer [8], using urease inhibitors [9], planting highly efficient rice varieties [10], and combined organic and inorganic fertilizer applications [11]. However, higher prices (controlled released fertilizer), related knowledge requirements (chlorophyll reading) or extra labour inputs (topdressing) have restricted the spread of these technologies. In addition, labour prices have increased year by year because farmers have got older and people have left the land. This has meant that both farmers and the government are more willing to accept simplified fertilization patterns, such as one—time fertilization, as long as they do not lead to yield reductions. Fertilizer application times and rates play key role in influencing plant growth and nutrient uptake [12]. Therefore, identifying the optimum fertilization time and rate have become extremely important for one—time fertilization programs.

Fertilizer—N spatial distribution and plant N absorption are affected by N forms and soil types. Previous research has shown that N translocation of ammonium sulphate mainly occurred in the 0–5 cm soil layer when the fertilizer rate was three times the conventional rate [13]. The mobility of fertilizer—N is faster in sandy soils than in loamy soils. As a result, the losses caused by leaching and runoff from sandy soils can be considerable [14]. Beek et al. [15] showed that N leaching in sandy soils was about 73 kg N ha^–1^ year^–1^ on grassland, which was significantly higher than for clay soils (15 kg N ha^–1^ year^–1^). Therefore, careful fertilizer management, where low doses are applied as a number of split applications over the growing season, is recommended for sandy soil. RZF has been reported to significantly decrease NH_3_ volatilization [16], reduce fertilizer—N surface runoff [16], prevent nitrous oxide and nitric oxide emissions from nitrification and denitrification [17], and increase grain yield and nutrient use efficiency [18]. The effects of a one—time RZF application on rice have been reported in South and Southeast Asia [19, 20]. However, little systematic research has been carried out on paddy rice in China. The Chinese government wishes to lower N application rates, reduce N loss and enhance NUE as much as possible. Site—specific N management and controlled release fertilizer application could enhance NUE from 31 to 40% and from 38 to 57%, respectively, without yield reduction [5, 8]. However, information about RZF improvements to NUE and N loss reductions is limited. Few studies have examined the fertilizer—N fate of one—time RZF in different soil types. Therefore, our understanding of the N transportation and plant uptake characteristics of one—time RZF treatments needs to improve. The objectives of this research were to investigate the effect of different fertilization patterns (FFP and RZF) on rice yield, fertilizer—N diffusion kinetics, N uptake and NUE using the ^15^N tracer technique in the Yangtze River basin, one of the main rice planting regions of China.

## Materials and Methods

### Field experiment

**Fig 1:**
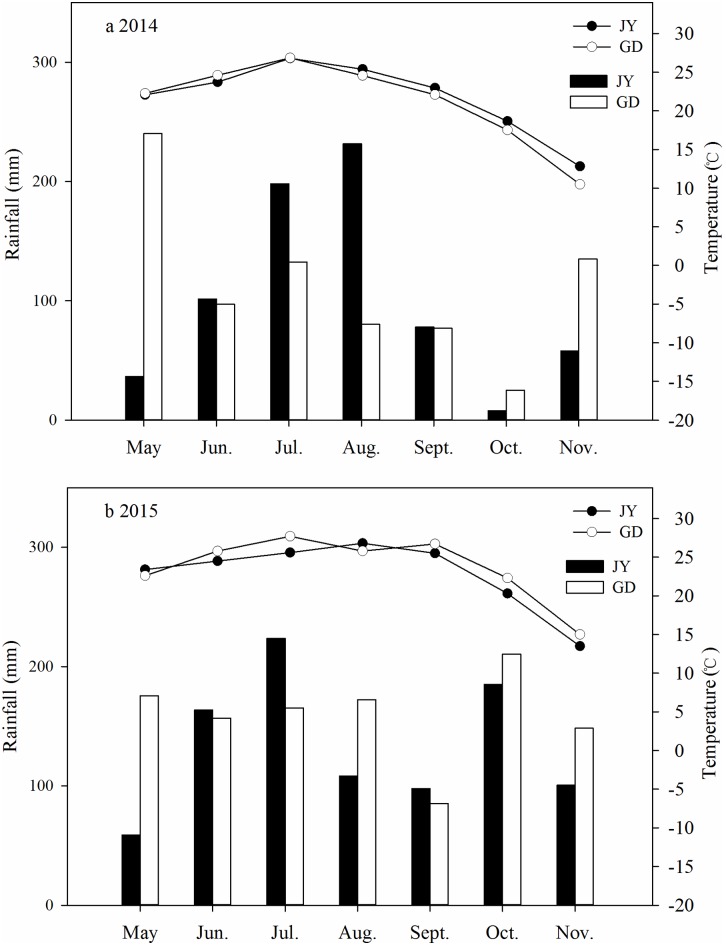
Rainfall (bars) and average temperatures (lines) in Guangde (GD) and Jiangyan (JY) from May to November during 2014 (a) and 2015 (b).

On—farm experiments were carried out with two different soil types in 2014 and 2015. The first site was in Guangde County, Xuancheng City, Anhui Province, China (GD) (31°01′44.87″N, 119°27′42.00″E). The other was in Jiangyan County, Taizhou City, Jiangsu Province, China (JY) (32°26′10.83″N, 120°05′43.28″E). Soil physicochemical properties and meteorological data for two sites are shown in Table 1 and Fig 1, respectively. Soil pH was measured in 1:1 (v/v) soil to water ratio using a pH meter. Soil bulk density was measured by core cutter method. Soil total N, total P and total K content were measured by Kjeldahl method, colorimetric analysis and flame photometry, respectively. Soil organic matter was determined by the wet combustion method. Soil particle composition was determined by hydrometer method [21].

**Table 1:** Physicochemical properties of the experimental soils from the two experimental sites in 2014 and 2015.

Year	Site	pH	Total N (g kg^–1^)		Soil bulk density (g cm^3^)		Total P (g kg^–1^)	Total K (g kg^–1^)	Organic matter (g kg^–1^)	HNO_3_–K (mg kg^–1^)	Available K (mg kg^–1^)	Soil particle composition (%)			Soil texture
			0–20 cm	20–40 cm	0–20 cm	20–40 cm						2–0.05 mm	0.05–0.002 mm	<0.002 mm	
2014	Jiangyan	7.4	1.784	0.644	1.4	1.42	0.94	20.2	10.8	584.1	132.9	45	46	9	sandy loam
	Guangde	6.5	1.478	0.442	1.44	1.47	0.35	15.3	6.5	251.2	33.5	23	64	13	loam
2015	Jiangyan	7.6	1.622	0.663	1.38	1.41	0.99	23.2	11.5	505.5	169.3	42	44	14	sandy loam
	Guangde	7	1.556	0.427	1.43	1.45	0.52	17.4	7.6	421.7	61.6	21	61	18	loam

#### Experiment 1

The optimum growth stage for a one—time N application was obtained by undertaking a micro—plot experiment in GD county during 2014. The different N fertilization treatments were as follows:

1. CK: Control with no N fertilization;
2. FFP: N fertilizer (100 mg N kg^–1^ air dried soil) was split into three applications: 40% as basal fertilizer (mixed with soil), 30% as tillering fertilizer and 30% as jointing fertilizer (broadcast on the soil surface);
3. Twenty one different fertilization treatments. There were three N fertilization stages: at transplanting (basal fertilizer on June 20th), at the tillering stage (jointing fertilizer on July 15th) or at the booting stage (panicle fertilizer, on August 10th). Each stage included seven N rates applied at 30, 60, 90, 120, 150, 180 and 210 mg N kg^–1^ air dried soil.

The plots were prepared as follows: first, a hole (25 cm length, 18 cm width) was dug down until the soil below the plough layer was reached. A polyvinyl chloride (PVC) frame (25 cm length, 18 cm width and 35 cm height; open at the top and bottom) was placed in the hole. Then the frame was filled with 8 kg screened, air—dried soil to 20 cm depth. When the urea was applied as basal fertilizer, it was intensively mixed with the soil before the mix was put into the PVC frame. When the urea was applied as jointing or panicle fertilizer, some pinholes were drilled around the rice root and then the urea solution was uniformly poured into the pinholes. Each treatment was replicated eight times. The frames were randomly distributed across the open field. Three seedlings were transplanted into each PVC frame after irrigation.

#### Experiment 2

A 2–year—long field experiment was undertaken at both sites to evaluate the effect of fertilization patterns (FFP and RZF) on rice yield, fertilizer—N fate and NUE. Three urea application patterns were investigated:

1. CK: Control with no N fertilization;
2. FFP: Urea was split into three applications, 40% as basal fertilizer (ploughed into the soil tillage layer before flooding the field), 30% as tillering fertilizer and the remaining 30% as jointing fertilizer (urea was broadcast on the soil surface);
3. RZF: Urea applied once as basal fertilizer into 10 cm deep holes positioned 5 cm from the rice roots.

The N rate was 225 kg N ha^−1^ for both FFP and RZF at both sites. The procedure for the RZF treatment was as follows: before transplanting the rice, P and K were applied and then the field was irrigated with little water to muddy the soil. The rice was transplanted with 18 cm interplanting distance and 25 cm inter row spacing. After transplanting the plants into each plot, a 10 cm deep hole was dug between two plants (18 cm interplanting distance) using steel pipe (1 cm diameter, the pipe structure is similar to the injection syringe). The hole was 5 cm away from the main rice root. The inner piston of the pipe was pulled. The urea granule was placed in the pipe, which was carefully pressed to the bottom using the piston to avoid urea sticking to the wall. Then the pipe was removed and the hole quickly filled with mud. During this process, the operator walked backward in order to avoid disturbing the fertilization site.

Each plot area was 20 m^2^ and each treatment was replicated four times in a randomized block arrangement. To measure the transportation of fertilizer—N under the different fertilization patterns, four random rice plants from the centre of each plot (except CK) were chosen and surrounded by a plastic cube that had no top and bottom (length 18 cm, width 25 cm and height 35 cm). They were given ^15^N labelled urea (10.15% atom abundance, produced by the Shanghai Research Institute of Chemical Industry) instead of normal urea. The plastic cube was inserted into the soil to a depth of 20 cm, which meant that 15 cm remained above ground to avoid cross contamination.

### Plot management

For both experiments, 90 kg phosphorus pentoxide (P_2_O_5_) ha^−1^ (single superphosphate) and 120 kg dipotassium oxide (K_2_O) ha^−1^ (potassium chloride) were applied to the soil tillage layer 1 day before flooding the field. Rice seeds (*Oryza sativa* L., cv. *Wuyunjing 24*) were sown in germination pans containing 10 cm of compost with 1 cm of compost on top of the seeds. Seedlings with three leaves (appearing about 25–30 d after sowing) were transplanted into the field at a density of 22 hills m^−2^ (25 × 18 cm) and two plants per hill (Experiment 2). After transplantation, the plants were grown under waterlogged conditions (i.e. a layer of water about 4–5 cm above the soil surface was maintained). Each plot had independent drainage and irrigation ditches to prevent the spread of water and fertilizer between plots. Fungicide (phosethyl—Al) and insecticide (avermectin—hexaflumuron) were sprayed each month throughout the experiment.

### Sample and chemical analysis

**Fig 2:**
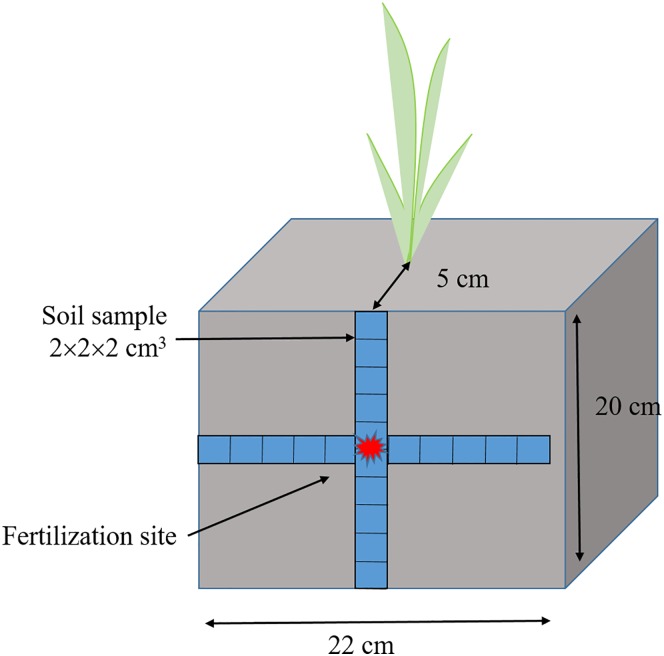
Diagram of soil sampling process. Red point means fertilizer site at 10cm depth. Each blue square means a 2×2×2 cm^3^ soil cube.

In Experiment 1, straw and grain were separately collected with eight replicates at the harvesting stage. In Experiment 2, ten rice plants in each plot were randomly sampled at the tillering, jointing, booting and harvesting stage (Table 2) to study the growth and N uptake differences between the fertilization patterns. The soils were sampled with four replicates at 30 d, 60 d and 90 d after rice transplanting in 2014 (Table 2) so that fertilizer—N movement and transportation could be investigated. Before the soil was sampled, a randomly selected rice plant was surrounded by a steel cube (length 18 cm, width 25 cm and height 35 cm). The cube was pressed into the soil to 20 cm depth to prevent water seepage. The water was drained from the inside of the steel cube. After the water had drained for two hours, a sharp shovel was used to carefully dig out a 20 cm depth section that was 7 cm away from the rice plant and on the same side as the fertilization treatment. Then a plastic plate was used to protect the section and to avoid collapse. The 2 × 2 × 2 cm^3^ soil cube, centred on the fertilization site, was sampled in—situ from left, to right, and from top to bottom on the section to determine NH_4_^+^–N content. In total, 10 subsections were in the horizontal direction and in vertical direction, respectively (Fig 2). Rice agronomic characters, including productive tillers per hill, spikelets per panicle and filled spikelets per panicle, were measured before harvest. Four rice plants from each plot that had been labelled with ^15^N urea were harvested together and separated into straw, grain and roots. The soil from the 0–20 and 20–40 cm layer in the plastic cube were collected, the roots were removed and the soils were pulverised and well blended. Finally, 1 kg of mixed soil from each soil layer in the plastic cube was sampled and stored until analyzed.

**Table 2:** Date of rice transplanting and sampling from the two experimental sites in 2014 and 2015.

Years and sites	Transplanting	Rice sample of tillering	Tiller fertilizer	Soil sample after 30 d	Rice sample of jointing	Jointing fertilizer	Soil sample after 60 d	Rice sample of booting	Soil sample after 90 d	Harvest
2014 Jiangyan	12–Jun	30–Jun	1–Jul	12–Jul	1–Aug	2–Aug	11–Aug	10–Sep	10–Sep	10–Oct
2014 Guangde	12–Jun	28–Jun	30–Jun	12–Jul	31–Jul	1–Aug	11–Aug	10–Sep	10–Sep	10–Oct
2015 Jiangyan	17–Jun	30–Jun	30–Jun	17–Jul	31–Jul	1–Aug	16–Aug	10–Sep	15–Sep	14–Oct
2015 Guangde	14–Jun	28–Jun	30–Jun	14–Jul	31–Jul	1–Aug	13–Aug	10–Sep	12–Sep	12–Oct

Fresh soil samples were extracted by the 2 M KCl and NH_4_^+^–N (mg kg^–1^ dry soil) was analysed using indophenol blue method with a discrete auto analyzer (SmartChem 200, Analyzer Medical System Inc., Guidonia, Rome, Italy). The oven—dried plant samples and air—dried soil samples were ground to pass through a 100–mesh sieve. The soil N and plant N were measured using the Kjeldahl method. The ^15^N atom percentage of the plant and the soil sample were analysed using an isotope mass spectrometer (Delta V Advantage, Thermo Fisher Scientific Inc., Waltham, MA, USA). The percentage and amount of N derived from fertilizer, the fate of the N fertilizer etc., were calculated according to Zhu [22].

The following equations were used:

Soil N derive from fertilizer amount = Soil total N content×Soil bulk density×Soil volume×Percentage of N derived from fertilizer

N use efficiency (Difference method) = (N accumulation of the fertilized treatment – N accumulation of CK) / N rate×100.

$$\text{N use efficiency }\left({}^{15}\text{N labelled method}\right)\;{=}^{15}\text{N uptake by rice }{/}^{15}\text{N input}\times 100.$$

### Statistical analysis

All of the results are shown as the mean value of four or eight replicates. The experimental data were log—transformed if necessary to improve normality and homoscedasticity. A one—way ANOVA (for experiment 1) and a three—way ANOVA (for experiment 2) were undertaken to assess differences among treatments using SPSS v. 20 statistical software (IBM, Chicago, Illinois, USA). The NH_4_^+^–N transportation characteristics were described by Surfer 8.0 (Surface mapping system, Golden software. Inc. USA). Significant differences (*P* < 0.05) between treatments are indicated by different letters.

## Results

### Effect of N application stage and rate on rice yield and N uptake (Experiment1)

**Fig 3:**
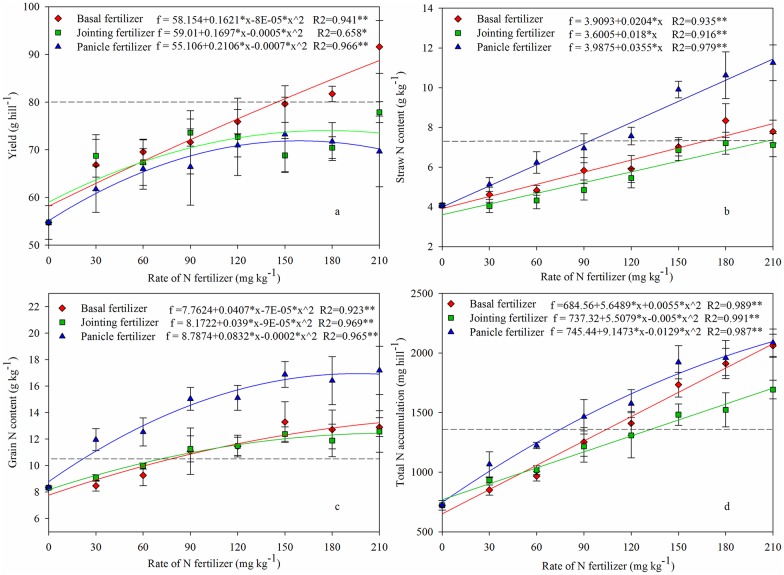
Effect of N application stage and N rate on rice Yield (a), Straw N content (b), Grain N content (c) and Total N accumulation (d). Horizontal dotted line is the value for the farmer fertilizer practice (FFP). * and ** means correlation is significant at the *P ≤* 0.05 and *P ≤* 0.01 level. (Experiment 1, 2014).

Rice yield increased as the N rate rose at each stage (Fig 3a). There was a good quadratic relationship between yield and N applied as a basal fertilizer (R^2^ = 0.941**), and the yield increment order as the N rate rose was basal fertilizer > jointing fertilizer > panicle fertilizer. The highest yield (91.6 g hill^–1^) was observed when 210 mg N kg^–1^ soil was applied as basal fertilizer, which was a 14.6% improvement on FFP (79.9 g hill^–1^). However, treatment yields when N was applied as a jointing or panicle fertilizer were all lower than FFP regardless of N rate.

The grain and straw N content showed a positive correlation with N rate, and the R^2^ values ranged from 0.916 to 0.979 (Fig 3b and 3c). N applied as a panicle fertilizer significantly increased the N content of the grain and straw compared to the basal and jointing fertilizer at the same N rate. Both grain and total N accumulation were significantly higher when urea was applied as a panicle fertilizer than as a jointing or basal fertilizer, except for 210 mg N kg^–1^ soil (Fig 3d).

### Effect of fertilization pattern on soil NH_4_^+^–N (Experiment 2)

**Fig 4:**
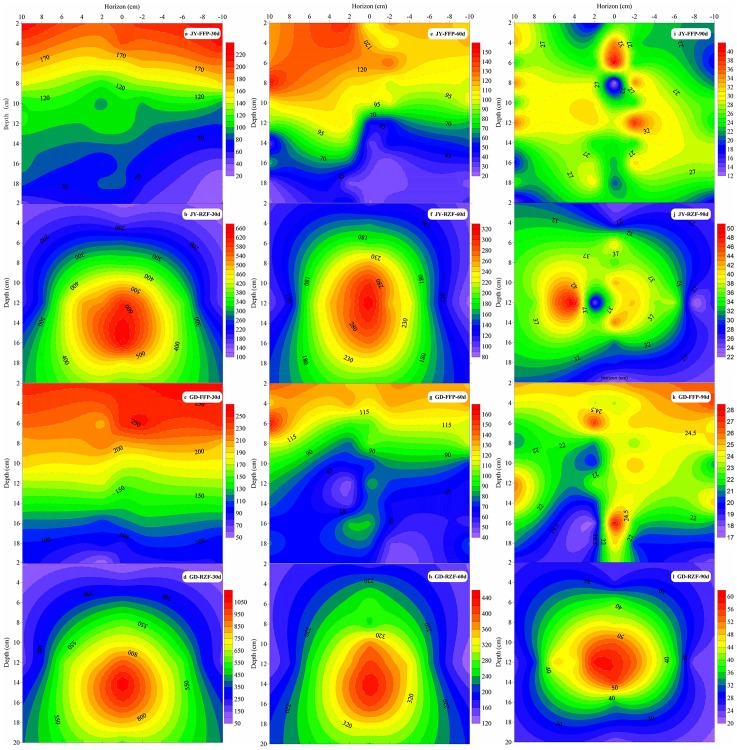
Effect of fertilization pattern on soil NH_4_^+^-N content (mg kg^-1^ dry soil). NH_4_^+^-N content of the different soil layers at 30 d (a-d), 60 d (e-h) and 90 d (i-l) after transplanting, respectively. GD and JY refer to Guangde county and Jiangyan county, respectively. For the FFP treatment, the urea was split into three different applications: 40% as basal fertilizer (ploughed into the soil tillage layer before flooding the field), 30% as tillering fertilizer and the remaining 30% as jointing fertilizer (urea was broadcast on the soil surface). For the RZF treatment, the urea was applied once into 10 cm deep holes that were positioned 5 cm from the rice roots as basal fertilizer. (Experiment 2, 2014).

The results showed that only NH_4_^+^–N content was significantly affected by the N fertilization patterns in paddy soil. Therefore, only NH_4_^+^–N content is shown (Fig 4). For the FFP, the maximum NH_4_^+^–N value occurred at the soil surface and it declined as the soil depth increased. The maximum average NH_4_^+^–N value for FFP at the two sites after 30 d (Fig 4a and 4c), 60 d (Fig 4e and 4g) and 90 d (Fig 4i and 4k) were 219.9, 114.1 and 25.3 mg kg^–1^, respectively. However, for the RZF regime, NH_4_^+^–N continuously rose as the soil depth increased and the maximum value was observed at 10–14 cm soil depth. NH_4_^+^–N content in the RZF plots decreased as time progressed. The maximum average NH_4_^+^–N value for the RZF regime at the two sites after 30 d (Fig 4b and 4d), 60 d (Fig 4f and 4h) and 90 d (Fig 4j and 4l) were 861.8, 369.9 and 50.2 mg kg^–1^, respectively. Furthermore, for the RZF treatment, NH_4_^+^–N content at GD was higher than at JY under the same treatment.

### Effect of fertilization pattern on rice growth (Experiment 2)

An analysis of variance of the two years results from Experiment 2 (Table 3) showed that all the main factor effects (except Year × Site) on grain yield were significant (*P* < 0.01). Most of the agronomic characters, and N content and accumulation, were significantly (*P* < 0.01) affected by single and multiple factors. However, the fertilizer—N fate was not significantly affected by site, year × site and year × treatment.

**Table 3:** Analysis of variance results for dry weight, agronomic characteristics, N content, N accumulation, fertilizer N fate and NAE for experiment 2 (*P*–value).

Source of variation	Dry weight			Agronomic characters				N content			N accumulation				Fertilizer—N fate			NAE
	Grain	Straw	Root	Productive tiller per hill	Spikelets per panicle	Filled spikelets	1000 grain weight	Grain	Straw	Root	Grain N accumulation	Straw N accumulation	Root N accumulation	Total N accumulation	Rice uptake	Residual	Loss	
Year	<0.01	<0.01	<0.01	<0.01	<0.01	<0.01	<0.01	<0.01	<0.01	0.029	<0.01	0.267	<0.01	<0.01	<0.01	0.838	<0.01	<0.01
Site	<0.01	<0.01	<0.01	<0.01	0.060	0.311	<0.01	<0.01	<0.01	<0.01	<0.01	0.031	0.029	<0.01	0.359	0.037	0.099	0.356
Treatment	<0.01	<0.01	<0.01	<0.01	<0.01	<0.01	<0.01	<0.01	<0.01	<0.01	<0.01	<0.01	<0.01	<0.01	<0.01	<0.01	<0.01	<0.01
Year * Site	0.551	0.031	0.020	0.209	<0.01	<0.01	<0.01	<0.01	<0.01	<0.01	0.014	<0.01	<0.01	<0.01	0.086	0.250	0.675	<0.01
Year * Treatment	<0.01	<0.01	0.014	<0.01	0.023	0.356	<0.01	<0.01	<0.01	0.655	<0.01	<0.01	0.050	<0.01	0.179	0.787	0.443	<0.01
Site * Treatment	<0.01	<0.01	0.149	0.098	<0.01	<0.01	<0.01	<0.01	<0.01	<0.01	0.013	0.452	0.229	0.035	0.119	<0.01	<0.01	0.078
Year * Site * Treatment	<0.01	<0.01	<0.01	<0.01	<0.01	<0.01	0.095	<0.01	<0.01	<0.01	<0.01	<0.01	<0.01	<0.01	<0.01	<0.01	0.494	<0.01

**Fig 5:**
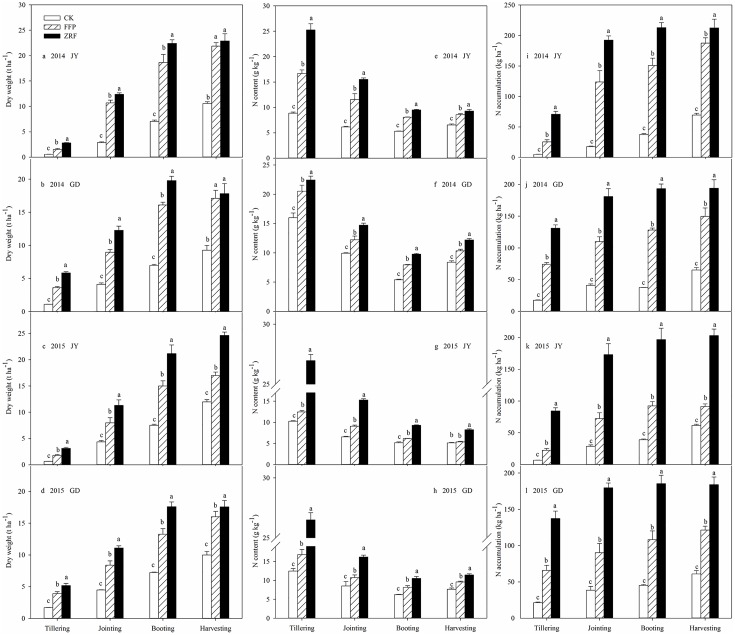
Effect of fertilization pattern on rice growth and N uptake at different stages. Dry weight (a–d). N content (e–h). N accumulation (i–l). GD and JY refer to Guangde county and Jiangyan county, respectively. CK, no N fertilizer. For the FFP treatment, the urea was split into three different applications: 40% as basal fertilizer (ploughed into the soil tillage layer before flooding the field), 30% as tillering fertilizer and the remaining 30% as jointing fertilizer (urea was broadcast onto the soil surface). For the RZF treatment, the urea was applied once into 10 cm deep holes that were positioned 5 cm from the rice roots as basal fertilizer. Different lowercase letters indicate significant differences (*P* < 0.05) between treatments with the same growth stage. (Experiment 2, 2014).

The results showed that RZF almost produced the highest plant dry weight at the booting stage, and it was significantly higher (average 28.4%) than FFP at both sites (*P* < 0.05) over the two years (Fig 5a–5d). A significantly higher N content (*P* < 0.05) was observed in RZF than in FFP at all growth stages (Fig 5e–5h). RZF led to maximum N accumulation earlier (at the jointing stage) than FFP (at the harvesting stage). The average N accumulation in the RZF plots at the tillering, jointing, booting and harvesting stages were 125.6, 83.2, 64.5 and 44.3%, respectively, which were than the levels recorded in the FFP plots (Fig 5i–5l). The productive tiller numbers in the RZF plots increased by 15.2–60.9% (*P* < 0.05) compared to FFP at both sites and for both years (Table 4). The other agronomic characteristics showed diverse changes due to different growing environments. RZF improved the grain dry weight compared to FFP at both sites (JY 8.3% and GD 3.4% in 2014; JY 44.8% and GD 9.0% in 2015). However, these improvements were not significantly different (*P* > 0.05) in 2014. RZF significantly increased grain N content, straw N content, root N content, grain N accumulation, straw N accumulation, root N accumulation and total N accumulation (*P* < 0.05) compared to FFP, and the average increases at the two sites over the two years were 18.1, 55.2, 33.3, 38.5, 83.4, 40.8 and 50.9%, respectively. The total N accumulations for RZF were 229.0 kg ha^–1^ (2014, JY), 218.5 kg ha^–1^ (2014, GD), 236.6 kg ha^–1^ (2015, JY) and 210.7 kg ha^–1^ (2014, GD), respectively, which were significantly higher than FFP and almost equal to the fertilizer N input (225 kg ha^–1^).

**Table 4:** Effect of fertilization pattern on rice agronomic characteristics, dry weight, N content and N accumulation at the two sites.

Year	Site	Treatment	Agronomic characters				Dry weight (t ha^–1^)			N content (g kg^–1^)			N accumulation (kg ha^–1^)			
			Productive tillers per hill	Spikelets per panicle	Filled spikelets per panicle	1000 grain weight (g)	Grain	Straw	Root	Grain	Straw	Root	Grain	Straw	Root	Total
2014	Jiangyan	CK	6.9c	148b	147a	28.2a	6.0b (49.6)	4.6a (38.0)	1.5b (12.4)	8.3c	4.3b	7.1c	49.5c (61.7)	19.9b (24.9)	10.7b (13.4)	80.1c
		FFP	12.9b	193a	151ab	25.5b	11.6a (48.9)	10.2a (43.0)	1.9a (8.0)	10.1b	6.9a	8.3b	117.1b (57.6)	70.4a (34.7)	15.6a (7.7)	203.2b
		RZF	14.9a	176ab	138b	28.6a	12.6a (51.0)	10.3a (41.7)	1.8a (7.3)	10.9a	7.3a	9.1a	137.0a (59.8)	75.6a (33.0)	16.5a (7.2)	229.1a
	Guangde	CK	7.4c	106c	104b	28.5a	5.6b (53.3)	3.7b (35.2)	1.2b (11.4)	8.4c	4.9c	6.4c	47.1c (64.7)	18.0c (24.8)	7.7c (10.5)	72.8c
		FFP	13.6b	172b	132a	27.5b	9.8a (51.9)	7.3a (38.6)	1.8a (9.5)	10.3b	6.6b	9.1b	101.7b (61.4)	47.9b (29.0)	15.9b (9.6)	165.6b
		RZF	17.8a	188a	135a	24.5c	10.3a (52.8)	7.3a (37.4)	1.9a (9.7)	12.2a	9.1a	14.1a	125.1a (57.4)	66.3a (30.2)	27.1a (12.4)	218.5a
2015	Jiangyan	CK	5.5c	102c	100c	28.0a	6.0c (43.5)	6.1c (44.2)	1.7c (12.3)	7.9b	3.2c	6.7c	46.5c (60.0)	19.2c (24.9)	11.7c (15.1)	77.4c
		FFP	10.3b	154b	147a	26.3b	8.5b (44.0)	8.5b (44.0)	2.3b (11.9)	7.3c	3.5b	8.7b	61.8b (55.4)	29.7b (26.7)	19.9b (17.9)	111.5b
		RZF	16.5a	121a	111b	25.7c	12.2a (44.5)	12.4a (45.3)	2.8a (10.2)	9.3a	7.2a	11.6a	113.8a (48.3)	89.4a (38.0)	32.3a (13.8)	235.6a
	Guangde	CK	8.5c	135b	127b	21.9b	5.1c (44.3)	4.9c (42.6)	1.5b (13.0)	7.7c	4.5c	7.1c	39.0c (54.8)	21.9c (30.8)	10.3c (14.4)	71.2c
		FFP	11.4b	150b	125b	24.5a	7.9b (43.4)	8.1b (44.5)	2.2a (12.1)	9.6b	5.6b	9.4b	75.7b (53.0)	45.8b (32.1)	21.2b (14.9)	142.7b
		RZF	14.8a	182a	153a	16.0c	8.6a (43.7)	9.0a (45.7)	2.1a (10.7)	11.4a	9.5a	12.6a	98.4a (46.7)	85.8a (40.7)	26.6a (12.6)	210.7a

CK, no N fertilizer. FFP, farmer fertilization practice. RZF, one—time root—zone fertilization. Values are the mean of four replicates. Different lowercase letters indicate significant differences (*P* < 0.05) between treatments within the same experimental site, according to the LSD tests. Values in parentheses indicate the percentage accounted for by total dry weight accumulation and N accumulation.

### Effect of fertilization pattern on the fate of fertilizer N (Experiment 2)

The percentage N derived from fertilizer (N_dff_%) in the 0–40 cm soil layer, and from the grain, straw and roots of the plants in the RZF plots were significantly higher than for FFP at both sites (Table 5). The average N_dff_% values for the grain, straw and root samples from the RZF plots were 45.7, 44.2 and 35.8%, respectively, significantly (*P* < 0.05) higher than FFP at the two sites over the two years. The amount of N derived from fertilizer (N_dff_ kg ha^–1^) showed similar changes to N_dff_%. Both N_dff_ (kg ha^–1^) for the 0–40 cm soil layer, and the grain, straw and root samples from the RZF plots were significantly (*P* < 0.05) higher than for FFP at both sites, and the average increase caused by RZF ranged from 87.3 to 250.3%.

**Table 5:** Effect of fertilization pattern on fertilizer N fate.

Year	Site	Treatment	Percentage of N derived from fertilizer (%)					Amount of N derived from fertilizer (kg ha^–1^)					Fate of fertilizer N (kg ha^–1^)			N use efficiency (%)	
			Soil 0–20cm	Soil 20–40 cm	Grain	Straw	Root	Soil 0–20cm	Soil 20–40 cm	Grain	Straw	Root	Rice uptake	Remains in soil	Loss	Difference method	^15^N labeled method
2014	Jiangyan	FFP	0.76b	0.17b	21.6b	21.6b	15.9b	37.9b	3.2b	25.3b	15.2b	2.5b	42.9b (19.1)	41.1b (18.3)	140.9a (62.6)	54.6b	19.1b
		RZF	1.49a	0.27a	45.9a	45.0a	31.0a	74.6a	3.9a	62.7a	33.9a	5.1a	101.8a (45.2)	78.5a (34.9)	43.7b (19.9)	66.2a	45.2a
	Guangde	FFP	1.11b	0.13b	18.9b	18.9b	16.2b	47.1b	1.7b	19.2b	9.1b	2.6b	30.9b (13.7)	48.7b (21.7)	145.4a (64.6)	41.2b	13.7b
		RZF	2.03a	0.40a	50.0a	48.8a	45.2a	86.3a	5.2a	62.6a	32.2a	12.3a	107.1a (47.6)	91.6a (40.7)	26.3b (11.7)	64.8a	47.6a
2015	Jiangyan	FFP	1.03b	0.18b	23.7b	24.1b	16.2b	46.0b	3.4a	14.7b	7.2b	3.2b	25.1b (11.1)	49.4b (22.0)	150.5a (66.9)	15.2b	11.1b
		RZF	1.38a	0.19a	41.1a	40.7a	33.3a	61.7a	3.6a	46.8a	36.4a	10.7a	93.9a (41.7)	65.4a (29.1)	65.7b (29.2)	70.3a	41.7a
	Guangde	FFP	0.67b	0.20b	20.9b	25.1b	16.7b	29.8b	2.5b	15.8b	11.5b	3.6b	30.9b (13.7)	32.3b (14.3)	161.9a (71.5)	31.8b	13.7b
		RZF	1.57a	0.68a	45.8a	42.1a	33.9a	69.8a	8.4a	45.1a	36.1a	9.0a	90.2a (40.1)	78.2a (34.8)	56.6b (25.1)	62.0a	40.1a

FFP, farmer fertilization practice. RZF, one—time root—zone fertilization. Values are the mean of four replicates. Different lowercase letters indicate significant differences (*P* < 0.05) between treatments within the same site, according to the LSD tests. Values in parentheses indicate the percentage accounted for the total N input.

After harvesting, the percentage fertilizer N absorbed by rice in the RZF plots ranged from 40.1 to 47.6%. This was significantly (*P* < 0.05) higher than for FFP, which only ranged from 11.1 to 19.1%. The change trend for the fertilizer N that remained in the 0–40 cm soil layer was similar for both sites. Between 29.1 and 40.7% of the fertilizer N remained in the 0–40 cm soil layer of the RZF plots, but only 14.3–22.0% remained in the FFP plots (markedly lower than RZF) at the two sites over the two years. The data showed that the highest percentage fertilizer—N loss was 29.2% for RZF, and this occurred at JY during 2015, whereas, at least 62.6% of the fertilizer—N in the FFP plots was lost over the two years. The N apparent recovery efficiency for RZF when calculated by the difference and labelled methods ranged from 62.0–70.3% and 40.1–47.6% respectively, which were significantly (*P* < 0.05) higher than for FFP at both sites.

## Discussion

### Optimal fertilization stage and pattern

In Experiment 1, when N applied once as basal fertilizer (urea homogeneously mixed with topsoil), a significant quadratic relationship was observed between N rate and rice yield (Fig 3a). However, the highest yield was observed at an N rate of 210 mg N kg^–1^ soil, which was significantly higher than when N was applied at any other stages and for FFP. Blending N with whole volumes of topsoil may show a priming effect for soil indigenous—N, and the uniform pre—treatment for each of the different fertilization stages at least demonstrated that there was a higher yield growth potential when large amount of N were applied as basal fertilizer compared to N applied at other stages. Therefore, one—time fertilization should focus on basal fertilizer applications. It has been reported that high nutrient concentration in the crop root—zone can increase root proliferation and enhance crop yield [23]. However, for convenience, rice basal fertilizer has traditionally been broadcast or mixed in the shallow soil layer by farmers, which just enhanced the NH_4_^+^–N content in the surficial soil layer (Fig 4a and 4c) and the floodwater, and could lead to an increased N loss risk instead of increasing soil N content in the root zone [24]. So, the difference of N concentration between the treatments of 210 mg N kg^–1^ soil once applied as basal fertilizer and the FFP which the basal fertilizer rate is 40 mg N kg^–1^ soil may also be the main reason for the yield gaps rather than differences in the gross fertilizer input amount (Fig 3a). However, there is no need to maintain a high N concentration throughout the entire topsoil layer during the early growth stages, because the rice root is too small and plant N demand is too low (Fig 5i–5l) [25]. Although urea is quickly hydrolysed at the soil surface after fertilization, in the complicated submerged paddy system, the strongly reduced environment and negative soil colloids restrict urea hydrolysis when the urea is placed at a deeper depth in the soil. The first hydrolysed NH_4_^+^–N after fertilization is quickly adsorbed by soil particles around the fertilizer site. This further inhibits urea hydrolysis and NH_4_^+^–N diffusion [20]. Therefore, changing the N fertilization pattern from broadcast to deep placement significantly increased the nutrient content in the root—zone without increasing the fertilizer rate (Fig 4). Broadcasting the urea onto the soil surface or mixing it with topsoil led to high NH_4_^+^–N contents (>100 mg kg^–1^) in less than 60 days. However, RZF significantly increased NH_4_^+^–N content around the fertilization site (about 10 cm in diameter, Fig 4) and this effect lasted more than 60 days. The high levels of N that remained in the root—zone were able to meet rice demands during the early growth stage (Figs 4b, 4d and 5i–5l). The high N uptake at the jointing and booting stage (Fig 5i–5l) explained why the NH_4_^+^–N content sharply decreased between 60 d and 90 d. In conclusion, the fertilization application pattern should be given more attention and N deep placement should be recommended as the optimal fertilization pattern for paddy land.

### Rice yield and N uptake

The reason we chose urea application at 5 cm horizontal distance and at 10 cm soil depth as the optimal RZF fertilization pattern was that 80% of the rice root were distributed in the 0–20 cm plough layer because a hard plough pan restrained downward growth [24]. Furthermore, shallow applications of N fertilizer (e.g. basal fertilizer applications under FFP) or applying N fertilizer too close to the rice root increased the risk of NH_3_ volatilization and NH_4_^+^ toxicity, respectively [16]. Another consideration is that N translocation and transformation mainly occurs in the 0–5 cm soil layer [13] and NH_4_^+^, which is hydrolysed from urea, tends to move downwards in water (Fig 4). Therefore, we applied fertilizer N at 5 cm horizontal distance and 10 cm depth in the RZF experimental sites [16].

RZF significantly increased rice yield by an average of 16.6% over FFP because it significantly (*P* < 0.05) increased rice productive tiller numbers (Table 4). This increase can be mainly attributed to sufficient supplementation of NH_4_^+^–N by RZF that met the needs of the earlier growth stages (Fig 4b and 4d) [19]. Many studies have shown that high basal N fertilizer applications lead to higher tiller numbers, aboveground biomass, and panicle and spikelet numbers, which have a positive relationship with yield [20, 26]. However, the changes in panicle numbers and 1000–grain weights at the two sites in 2014 were the opposite of 2015, which may be attributed to the different growth environments. Xiang et al.[27] reported that the N absorption rate is high during the jointing to booting stage for rice, and adequate N supply during this crucial stage plays a key role in improving rice yield. The plant must acquire 144 kg N ha^–1^ in the first 35 days following transplanting to achieve a high yield [28]. This is another explanation for the higher yields of RZF. The rice plants were able to absorb more N at the tillering stage under RZF than FFP (Fig 5i–5l). Although the average yield increase after RZF was higher than for FFP, there was no statistical difference between the fertilization patterns at both sites in 2014. Only an extremely significantly increase (44.8%) was observed at JY in 2015 (Table 4). Previous studies demonstrated the significant effects of environmental factors on plant yield in long term field experiments [29]. Therefore, the large variation in yield was mainly caused by climatic factors. Because the varieties, farming management regimes, and the soil conditions over the two years were the same at each site. In our experiment, we found that the large yield variation between RZF and FFP was mainly caused by variations in the FFP yields (Table 4). For example, the FFP yield at the JY site decreased from 11.6 t ha^–1^ in 2014 to 8.5 t ha^–1^ in 2015, while the yield of RZF only decreased from 12.6 to 12.2 t ha^–1^. The same change trend was observed at the GD site. Furthermore, the grain N content and biomass during the early growth stages (Fig 5) showed the same trend. This strongly indicated that RZF had an increased capacity to resist environmental risk compared to FFP.

It has been demonstrated that pre—anthesis N accumulation and N translocation are very important in improving the grain yield, because most grain N is transferred from the straw or blade to the grain [30]. Nearly 70% of the N absorbed by the straw will be translocated to the grain and this maintains grain N contents at a certain level during plant ripening. Nitrogen absorbed by rice during the vegetative growth stage contributes to growth during the reproductive and grain filling stages via translocation [31]. This study showed that rice N content and N accumulation of RZF were significantly higher (*P* < 0.05) than for FFP during the early growth stages (Fig 5), and that explained the high N accumulation in the stems, grain and roots at the harvest stage (Table 4). It has been reported that deep placement could reduce N fertilizer applications by 28–44% without leading to yield reduction [19]. However, although our results indicated that 29.1–40.7% fertilizer—N remained in the 0–40 cm soil layer of the RZF plots (Table 5), we suggest that the N rate could be cautiously reduced to avoid rapid reductions in soil fertility. This can be achieved because average total N uptake in the RZF plots (223.5 kg ha^–1^) was almost equal to the N input (Table 4). Therefore, further research should be undertaken to establish the optimum N rate for RZF regimes in the main rice production region of China.

### N allocation and NUE

Ammonia volatilization is considered to be the main cause of N loss in rice fields [32] and amounts to 18–38% of all fertilizer—N losses in a paddy field [6]. Rochette et al. [16] showed that the NH_4_^+^–N concentration in the surface water was the dominant factor affecting NH_3_ volatilization, and incorporating urea to depths > 7.5 cm resulted in negligible NH_3_ emissions and maximum N retention. The same results were observed in our research. The soil NH_4_^+^–N content was higher around fertilizer site and was retained for longer after RZF compared to FFP (Fig 4). It also explains why the fertilizer—N loss after RZF was significantly lower than after FFP (Table 5). The other reasons for the reduced NH_3_ volatilization losses in the RZF plots were that RZF considerably increased rice tiller numbers and dry matter accumulation during the vegetative growth stages compared to FFP (Table 4, Fig 5a–5d). The vigorous rice plant growth and the denser canopy in the RZF plots not only required more N (Fig 5), but also reduced floodwater temperatures by preventing the sunlight from reaching the field surface [33].

Although some researchers demonstrated that about 38–69% of fertilizer—N is lost through nitrification and denitrification in different soils, which is significantly more than NH_3_ volatilization and losses attributed to pot experiment conditions [34], we hypothesised that the major fertilizer—N loss pathway was via NH_3_ volatilization for FFP, because there was no heavy rainfall or drainage during the experimental period [35]. In contrast, it is possible that nitrification and denitrification represented the major pathways for N loss after RZF (10 cm depth) rather than NH_3_ volatilization because of the oxidizing conditions in the rice rhizosphere [17]. In addition, rice emits a considerable amount of NH_3_ into the ambient atmosphere, especially when there is an excessive ammonium N supply [30, 32]. Although RZF significantly reduces fertilizer—N loss compared to FFP, different soil types showed different results. The relatively lower NH_4_^+^–N content in the 0–20cm soil layer during the early growth stages (Fig 4b and 4f) and the reduction in fertilizer—N that remained in the 0–40 cm soil layer at the harvest stage (Table 5) indicated that leaching was greater in the sandy loam soil (JY) compared to the loam soil (GD). Furthermore, the large amounts of fertilizer—N that remained in the topsoil may enhanced the leaching risk in the following wheat crop. Therefore, future research should investigate ways of preventing N loss when using RZF.

NUE calculated by the difference method is equal to the N apparent recovery efficiency (NAR). NUE calculated by the labelled method represents plant uptake N directly from fertilizer [22]. From our results, we calculated that the average NUE (by difference method) for FFP was 35.7% (Table 5). The low value was consistent with previous research [10]. RZF significantly enhanced rice NUE according to the difference method (1.8 times higher on average) and by the labelled method (3.0 times higher on average) (Table 5). The NUE value calculated by the difference method is always higher than the labelled method because of positive priming and substitution effects between fertilizer—N and soil—N caused by immobilization [35].

The Chinese government and some researchers use the NAR as a specific indicator to evaluate whether a given fertilization strategy is efficient. Wang et al. [36] proposed a new formula to reappraise fertilizer—N in—season use efficiency because the NAR method has some drawbacks. For example, NAR is susceptible to control plot effects and NAR does not reflect fertilizer—N loss, etc. They maintained that the fertilizer—N remaining in the soil should be deducted when N use efficiency was calculated, because it can be used by the following crops. The results produced by this study further verified their views that although the NAR for FFP reached 41.2% and 54.6% at the two sites, respectively in 2014, fertilizer only directly contributed 19.1% and 13.7% to rice uptake, respectively (Table 5). Only 20% remained in the soil and more than 60% of the fertilizer was lost. A higher NAR value does not mean higher fertilizer—N use efficiency. Therefore, we suggest that the more efficient fertilizer strategies need to emphasize reducing N losses from the remaining fertilizer—N as far as possible, and an appropriate fertilization pattern plays a vital role in achieving this aim.

One difficult issue for RZF that needs to be addressed is labour cost and efficiency compared to FFP when fertilizer is applied manually. Although RZF significantly improved rice yield and NUE, and reduced N loss compared to FFP, farmer prefer to split and topdressing N fertilizer due to its low price and application costs. Therefore, the widespread acceptance of RZF application or fertilizer deep placement will largely depend on the manufacture of a light, simple fertilization machine that reduces the manual labour requirement. Alternatively, pressure to adopt the strategy should be applied at the national level.

## Conclusions

These results clearly showed that RZF can significantly increase rice grain yield, N uptake and N use efficiency in irrigated rice cropping systems, which reduces pollution and topdressing times compared to the traditional farmer practice. RZF is a promising technique that can be adopted by farmers growing rice in irrigated schemes once a simple fertilization machine has been produced in China. However, future research needs to investigate the most appropriate urea application sites and fertilization levels for different soil types, determine the effects of a combined N.P.K root zone fertilizer application on rice growth, and to develop RZF fertilizer application equipment.

## Supporting Information

S1 File Supporting information about data. (XLSX)
